# Differential effects of renal denervation on skin and muscle sympathetic nerve traffic in resistant and uncontrolled hypertension

**DOI:** 10.1007/s10286-023-00927-z

**Published:** 2023-01-25

**Authors:** Raffaella Dell’Oro, Fosca Quarti-Trevano, Gino Seravalle, Cesare Cuspidi, Guido Grassi

**Affiliations:** 1 Department of Medicine and Surgery, Clinica Medica, University of Milano-Bicocca, Via Pergolesi 33, 20052 Monza, Italy; 2 Istituto Auxologico Italiano, Milan, Italy

**Keywords:** Renal denervation, Sympathetic nervous system, Resistant hypertension, Blood pressure, Heart rate

## Abstract

**Purpose:**

Renal denervation (RDN) exerts sympathoinhibitory effects. No information is available, however, on whether these effects have a regional or a more generalized behavior.

**Methods:**

In 14 patients with resistant hypertension (RHT, age 58.3 ± 2.2 years, mean ± SEM), we recorded muscle and skin sympathetic nerve traffic (MSNA and SSNA, respectively) using the microneurographic technique, before, 1 month, and 3 months after RDN. Measurements included clinic blood pressure (BP), heart rate (HR), 24-h BP and HR, as well as routine laboratory and echocardiographic variables. Ten age-matched RHT patients who did not undergo RDN served as controls.

**Results:**

MSNA, but not SSNA, was markedly higher in RHT. RDN caused a significant reduction in MSNA 1 month after RDN, with this reduction increasing after 3 months (from 68.1 ± 2.5 to 64.8 ± 2.4 and 63.1 ± 2.6 bursts/100 heartbeats, *P* < 0.05). This effect was not accompanied by any significant change in SSNA (from 13.1 ± 0.5 to 13.4 ± 0.6 and 13.3 ± 0.4 bursts/min, *P* = NS). No quantitative or, in some cases, qualitative relationship was found between BP and the MSNA reduction induced by RDN. No significant changes in various sympathetic markers were detected in the control group who did not undergo RDN and were followed for 3-months observation.

**Conclusions:**

These data provide the first evidence that RDN exerts heterogeneous effects on sympathetic cardiovascular drive, inducing a marked reduction in MSNA but not in SSNA, which appears to be within the normal range in this condition.These effects may depend on the different reflex modulation regulating neuroadrenergic drive in these cardiovascular districts.

## Introduction

Resistant uncontrolled hypertension (RHT) is characterized by a pronounced sympathetic overdrive, as confirmed by increased urinary normetanephrine levels, augmented renal sympathetic spillover, heightened muscle sympathetic nerve traffic (MSNA) values, and elevated low-to-high frequency ratio of the heart rate variability signal [1–6]. There is also evidence that the neuroadrenergic overactivity characterizing RHT can be, at least partially, favorably affected by bilateral renal nerve ablation (RDN), which can reduce MSNA, whole-body norepinephrine spillover, and restore the above-mentioned low-to-high frequency variability ratio to close to normal values [4, 7–14]. No data are available on whether the sympathoinhibitory effects of RDN have a regional or a more systemic distribution, the only exception being represented by evidence that RDN may lower in resistant hypertensive patients renal norepinephrine spillover [4, 14]. The present study was designed to determine whether the sympathomodulatory effects of RDN in patients with RHT are generalized to the whole cardiovascular system or whether they are rather confined to selected vascular beds. To this aim, we systematically performed microneurographic recordings of muscle as well as skin sympathetic nerve traffic (SSNA) before and at different times following RDN. A further aim was to investigate an additional unexplored issue, namely the behavior of skin sympathetic nerve traffic in RHT patients before undergoing the RDN procedure.

## Methods

### Study population

As previously reported [15], the study population of this investigator-generated study consisted of a group of true resistant essential hypertensives, based on lack of office and ambulatory blood pressure (BP) control, despite daily administration of at least three antihypertensive drugs at full daily dosage. They belonged to a group of patients which we recruited in a previous study aimed at determining the effects of RDN on MSNA at different time periods after the procedure [15]. Measurements of MSNA and SSNA (see below) were obtained in 14 out of the 29 patients who underwent bilateral renal denervation, and the protocol of investigation has been described in the original paper. The remaining 15 patients evaluated in the original study were not included in the present analysis because it was technically impossible to obtain SSNA recording, either at baseline prior the RDN procedure or at the two different time periods after RDN. Measurements were performed in three experimental sessions, the first before and the others 1 month and 3 months after the RDN procedure. The same measurements were also obtained in another group of ten resistant hypertensive patients, matched for age, sex, and severity of the disease who did not undergo RDN and thus served as controls. Measurements of SSNA and MSNA were also repeated in these patients after 1- and 3-month time intervals, maintaining the unmodified the antihypertensive drug regimen. Patients were excluded from the study if they had clinical conditions known to affect sympathetic cardiovascular function, such as congestive heart failure, coronary artery disease, atrial fibrillation or other major cardiac arrhythmias, diabetes mellitus, obesity, metabolic syndrome, valvular heart disease, obstructive sleep apnea, renal insufficiency, or failure or poor adherence to antihypertensive drug treatment [16]. Smokers (three in the RDN group and two in the control group) were asked to refrain from smoking in the 24 h before each study session. The study protocol was approved by the Ethics Committee of the IRCCS Istituto Auxologico Italiano (Milan, Italy). All subjects gave written consent for the study after being informed of its nature and purpose, and the subjects were studied on an outpatient basis.

### Measurements

Measurements included sphygmomanometric and beat-to-beat finger (Finapres, Ohmeda, Englewood, USA) systolic and diastolic blood pressure, heart rate [cardiotacograph triggered on R wave of an electrocardiogram (EKG) lead]. They also included routine laboratory measurements (hemoglobin, blood urea, plasma creatinine, plasma electrolytes, estimated glomerular filtration rate, etc.) and an echocardiographic evaluation of the left ventricular mass index, calculated by the Devereux formula and normalized to body surface area, whereas left ventricular ejection fraction was measured from the four-chamber apical projection using the product area times length [17, 18]. Color Doppler and pulse Doppler were used to measure mitral flow [early diastolic peak flow velocity (E wave) and late diastolic peak flow velocity (A wave)] and flow at the left ventricular outflow tract. Ambulatory BP monitoring was obtained the day before the sympathetic nerve traffic recording over 24 h by an oscillometric device (Spacelabs 90,207; Spacelabs) with the readings set at 15- and 20-min intervals during daytime (from 7.00 am to 11.00 pm) and nighttime (from 11.00 pm to 7.00 am) periods, respectively [1, 15]. The device was applied in the morning, and subjects were allowed to return home with the instruction to attend their usual activities and to come back to the hospital the following day for device removal. The cutoff values for 24-h BP normality were those reported by international guidelines, i.e., < 130/80 mmHg [19]. Multi-unit recordings of MSNA or SSNA were obtained through a tungsten microelectrode inserted into a peroneal nerve posterior to the fibular head, as reported previously [1, 7, 11, 15]. The nerve signal was amplified by 70,000, fed through a band-pass filter (700–2000 Hz), and integrated with a custom nerve traffic analyzer. Integrated nerve activity was monitored by a loudspeaker, displayed on a storage oscilloscope (model 511A; Tektronix, Beaverton, OR), and digitized, with a sampling frequency of 1000 Hz (Powerlab recording system model ML870 8/30, AD Instruments, NSM2153, Australia) [1–15]. For MSNA, the criteria were that (1) a weak electrical stimulation through the microelectrode induced an involuntary muscle contraction but not paresthesias, (2) tapping or passive stretching of the muscle supplied by the nerve caused afferent mechanoreceptive impulses, and (3) the recording consisted of spontaneous pulse-synchronous bursts that increased during held expiration [1, 7, 11, 15]. For SSNA, the criteria were that (1) electrical stimulation through the microelectrode induced skin paresthesias without concomitant muscle contraction, (2) light skin touching evoked afferent nerve impulses, and (3) tapping or passive stretching of the muscle supplied by the nerve did not elicit afferent mechanoreceptive impulses [20, 21]. Neurograms were accepted only if they did not show simultaneous SSNA and MSNA activity and if the signal-to-noise ratio was greater than three. Muscle sympathetic nerve activity was quantified over a 30-min period as bursts per 100 heartbeats, whereas SSNA was quantified as bursts per min, also over a 30-min period.

### Protocol and data analysis

In the 14 RHT patients undergoing RDN, three experimental sessions were performed. The first session was carried out 24–48 h before the procedure for RDN, whereas the other two sessions were performed 1 and 3 months after the procedure, respectively. In the ten patients with RHT not undergoing RDN and thus serving as controls, three experimental sessions were also performed, with 1- and 3-month time intervals between the initial session and the other two, respectively. In each experimental session, which was carried out in the morning after a light breakfast, patients in the lying position were fitted with an intravenous cannula and devices for measuring sphygmomanometric and finger BP, heart rate, and respiration rate. After a 30-min interval, a blood sample for routine laboratory measurements was drawn from the cannula. Thereafter, three sphygmomanometric BP measurements were performed and then the microelectrode was inserted into a peroneal nerve to obtain MSNA or SSNA, which was recorded together with finger BP, heart rate, and respiration rate during a 30-min period. The microelectrode was then repositioned in the peroneal nerve fascicles to obtain the sympathetic nerve activity (MSNA or SSNA) that was not obtain in the previous recording period. Also in this instance, finger BP, heart rate, and respiration rate were all recorded during a 30-min period. Each protocol step was separated from the following one by a 20-min interval. Bilateral renal denervation was carried out by employing a radiofrequency ablation catheter (Symplicity, Medtronic Ardian, Palo Alto, CA, USA) according to a protocol previously described [15]. Data were collected in a semi-dark and quiet room at a constant temperature of 20–21 °C, and were analyzed by a single investigator unaware of the experimental design and of the group and session assignment of each patient. Office, 24-h, and finger mean systolic and diastolic BP; office, 24-h, and finger mean heart rate, respiration rate, resting MSNA, and SSNA obtained from individual patients were averaged separately for each group and each experimental session, and expressed as means ± SEM. In the group of patients who underwent RDN and in the control group, comparisons between data obtained in the different experimental sessions were made by one-way ANOVA for repeated measurements. Bonferroni correction was used for post-hoc analysis. Spearman analysis was used to correlate changes in different variables. A value of *P* < 0.05 was taken as statistical significance. References values for normal MSNA and SSNA were taken from a previous study in which a group of healthy normotensive subjects of a similar age was investigated [22].

## Results

### Baseline values

As presented in Table 1, the group of resistant hypertensive patients undergoing RDN and the resistant hypertensive control group had a similar age, gender distribution, routine laboratory values, echocardiographic parameters, clinic and 24-h ambulatory BP, and heart rate values, as well as antihypertensive drug treatment. In both groups, the MSNA values were two times greater than those found in the pure normotensive age-matched control subjects (MSNA 36.8 ± 5.7 bursts/100 heartbeats) [22]. In contrast, RHT showed SSNA values almost identical to those in age-matched healthy normotensive controls (SSNA: 12.7 ± 1.7 bursts/min) [22].

**Table 1:** Demographic, anthropometric, hemodynamic, clinical, and sympathetic nerve traffic values recorded at baseline in resistant hypertensive patients undergoing renal denervation (RHT–RDN) and in controls (RHT–control)

Variable	RHT–RDN	RHT–control
	(*n* = 14)	(*n* = 10)
Age (years)	58.3 ± 2.2	59.1 ± 2.6
Gender (males/females, *n*)	11/3	8/2
BMI (kg/m^2^)	26.7 ± 0.7	26.9 ± 1.0
Clinic blood pressure (S/D, mmHg)	169.4 ± 3.9/94.8 ± 3.4	166.1 ± 3.4/95.5 ± 3.3
Clinic heart rate (beats/min)	69.1 ± 3.5	67.5 ± 3.1
24-h blood pressure (S/D, mmHg)	160.1 ± 3.2/90.4 ± 3.0	159.5 ± 3.3/90.1 ± 3.2
24-h heart rate (beats/min)	65.4 ± 3.1	64.1 ± 2.9
LVEF (%)	60.2 ± 1.1	59.9 ± 1.2
LVMI (g/m^2^)	115.5 ± 3.9	114.3 ± 0.2
E/A ratio, a.u	0.99 ± 0.1	0.98 ± 0.1
eGFR (ml/min/1.73 m^2^)	79.3 ± 3.0	80.4 ± 3.4
Respiration rate (breaths/min)	17.4 ± 0.6	17.8 ± 0.7
MSNA (bursts/min)	45.6 ± 2.3	46.2 ± 2.8
MSNA (bursts/100 hb)	68.1 ± 2.5	70.3 ± 3.9
SSNA (bursts/min)	13.1 ± 0.5	13.8 ± 0.6
Anti-HT drugs (*n*/day)	4.4 ± 0.5	4.6 ± 0.7
ACEIs	74.0%	71.8%
ARBs	66.6%	63.5%
β-blockers	68.9%	69.3%
α-blockers	22.4%	24.4%
Calcium blockers	67.4%	69.1%
Diuretics	100.0%	100.0%
Centrally acting drugs	42.3%	44.8%
Aldosterone antagonists	45.8%	50.0%

Data are shown as means ± SEM. Clinic BP is the BP measured in each microneurographic experimental session. *S* systolic, *D* diastolic, *LVEF* left ventricular ejection fraction, *LVMI* left ventricular mass index, *eGFR* estimated glomerular filtration rate, *HT* hypertension, *MSNA* muscle sympathetic nerve traffic, *SSNA* skin sympathetic nerve traffic, *ACEI* angiotensin converting enzyme inhibitors, *ARB* angiotensin receptors blockers

### Effects of RDN

**Fig. 1:**
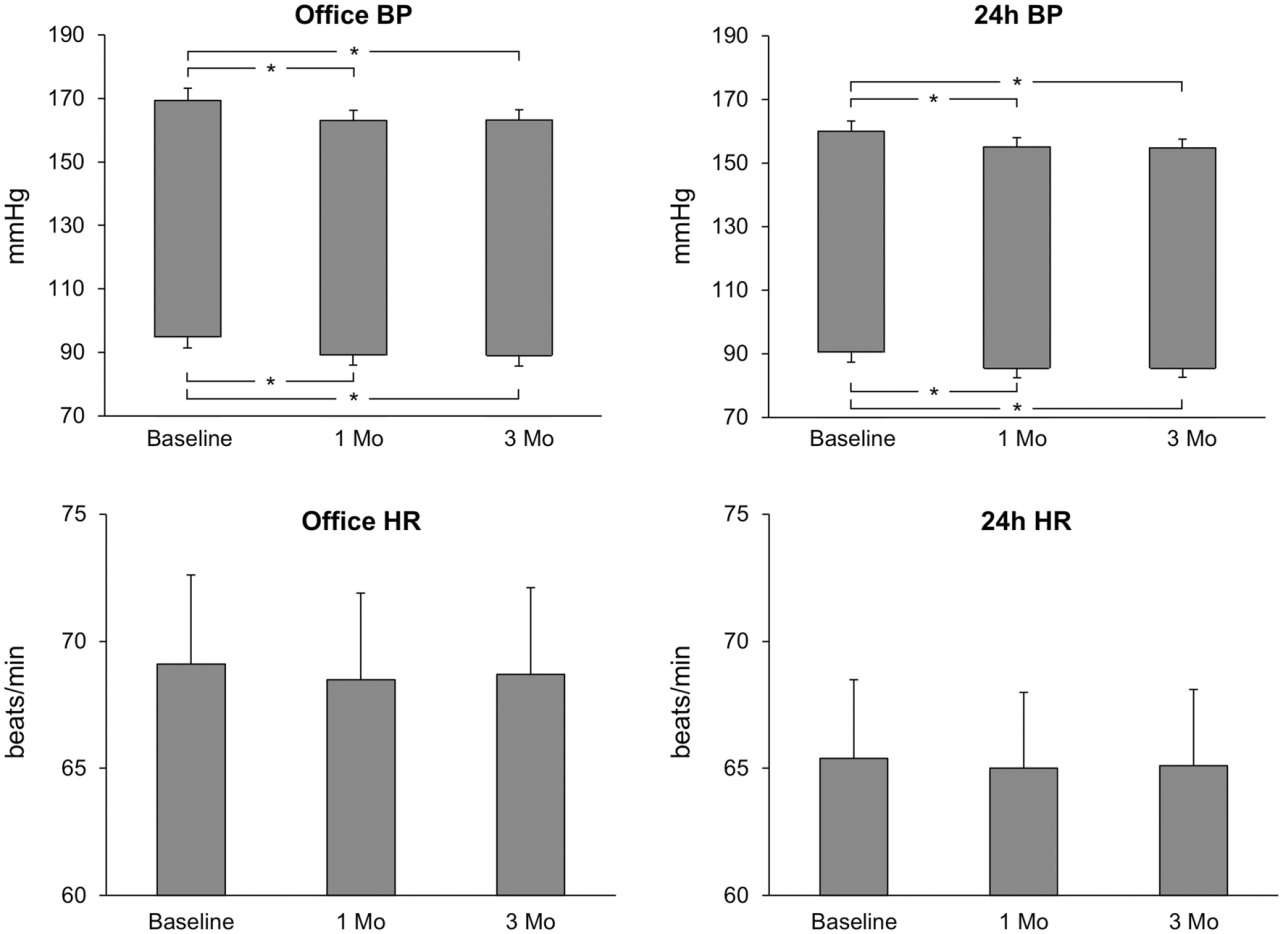
Effects of renal denervation on office blood pressure (BP), 24-h BP, office heart rate (HR), and 24-h HR values in resistant hypertensive patients. Data are represented before (baseline) and 1 and 3 months after the procedure. Data are shown as individual and average values (mean ± SEM) in each condition. Asterisks (*P < 0.05) refer to the statistical significance between baseline values and values recorded at different times after renal denervation

**Fig. 2:**
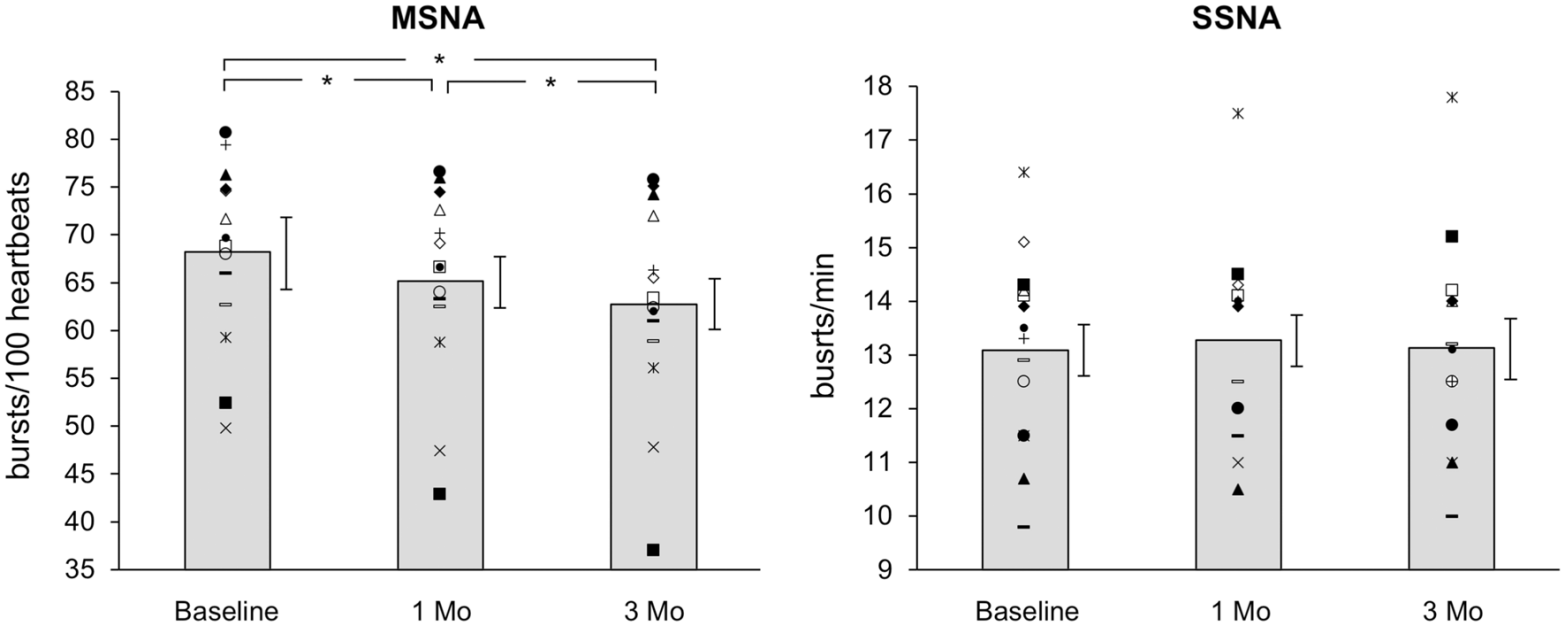
Effects of renal denervation on muscle sympathetic nerve traffic (MSNA, left panel) and skin sympathetic nerve traffic (SSNA, right panel) in resistant hypertensive patients. Data are shown before (baseline) and 1 and 3 months after the procedure. Data are shown as individual and average values (mean ± SEM) in each condition. Asterisks (*) refer to the statistical significance (*P* < 0.05) between baseline values and values recorded at different times after renal denervation

RDN was associated with a significant reduction in office and 24-h systolic and diastolic average BP values when evaluated 1 and 3 months after the procedure (Fig. 1, upper panels) but did not cause any significant change in either clinic or 24-h heart rate (Fig. 1, lower panels). Figure 2 shows the effect of RDN on individual and average MSNA and SSNA values (left and right panel, respectively). When assessed 1 month after RDN, MSNA values showed a significant reduction, which became greater in magnitude at the 3-month follow-up. This was in sharp contrast with the behavior of SSNA values, which remained unchanged 1 and 3 months after the procedure. No correlation was found between baseline pre-RDN office and 24-h BP values and the corresponding BP changes after the procedure. Similarly, no relationship was found between baseline MSNA values and MSNA changes after RDN, which were also unrelated to the clinic and 24-h BP changes. No significant relationship was found between clinic heart rate, 24-h heart rate changes associated with RDN and the concomitant MSNA and SSNA changes induced by the procedure. Routine laboratory values, including plasma electrolytes and estimated glomerular filtration rate were unchanged during post-RDN follow-up (data not shown), this also being the case for the echocardiographic parameters. The number and daily dosage of antihypertensive drugs remained unchanged in 12 patients, while in two patients there was a reduction in the daily dosage of spironolactone and beta-blocker metoprolol between the 1st and 3rd follow-up months.

### Control group

Table 2 reports the clinic BP, 24-h BP, clinic heart rate, 24-h heart rate, MSNA, and SSNA recorded at baseline and during the 3 months follow-up without RDN observed in the control group of resistant hypertensive patients. No significant change of any hemodynamic and sympathetic variable were observed at the 1st or 3rd month follow-up evaluation. This was the case also for the number and daily dosage of antihypertensive drugs administered.

**Table 2:** Hemodynamic and sympathetic variables detected at baseline and 1 and 3 months follow-up (FU) in the control group

Variable	Baseline	1-month FU	3-month FU
Clinic SBP (mmHg)	166.1 ± 3.4	169.4 ± 3.8	170.1 ± 3.8
Clinic DBP (mmHg)	95.5 ± 3.3	94.8 ± 3.5	96.1 ± 3.4
Clinic HR (beats/min)	67.5 ± 3.1	68.4 ± 2.8	68.0 ± 3.0
24-h SBP (mmHg)	159.5 ± 3.3	161.9 ± 3.2	163.5 ± 3.4
24-h DBP (mmHg)	90.1 ± 3.2	89.5 ± 3.1	91.3 ± 3.3
24-h HR (beats/min)	64.1 ± 2.9	67.2 ± 3.0	67.4 ± 2.8
Resp rate (breaths/min)	17.8 ± 0.7	17.4 ± 0.5	17.6 ± 0.7
Anti-HT drugs (*n*/day)	4.6 ± 0.7	4.7 ± 0.7	4.7 ± 0.8
MSNA (bursts/min)	48.2 ± 2.3	49.4 ± 2.4	49.9 ± 2.3
MSNA (bursts/100 hb)	70.3 ± 3.9	70.9 ± 3.8	72.8 ± 3.9
SSNA (bursts/min)	13.8 ± 0.6	14.5 ± 0.6	14.2 ± 0.5

Data are shown as means ± SEM. Clinic BP is the sphygmomanometric BP measured in each microneurographic experimental session. *SBP* systolic blood pressure, *DBP*diastolic blood pressure, *HR* heart rate, *Resp* respiration, *HT*hypertension, *MSNA* muscle sympathetic nerve traffic, *SSNA* skin sympathetic nerve traffic

## Discussion

Confirming previous evidence based on direct microneurographic recording of MSNA [7–11, 15], the present study shows that RDN exerts marked sympathoinhibitory effects, also confirming previous findings [7–11, 15], which do not appear to be quantitatively, and in some instances even qualitatively, related to the concomitant reductions in BP values. However, two major novel findings were identified. The first refers to evidence that the sympathoinhibitory effects of RDN do not appear to be uniformly distributed over the whole cardiovascular system, with the sympathetic deactivation detected in MSNA not being paralleled by a concomitant reduction in SSNA. From this, we conclude that the sympathetic deactivation associated with RDN is not uniformly distributed over the whole cardiovascular system. The second new finding refers to the evidence that RHT, although characterized by MSNA values which are two times greater than those found in pure normotensive age-matched individuals, has SSNA values almost identical to those seen in healthy subjects with normal blood pressure values. It thus appears that the hyperadrenergic state detected in resistant hypertensive patients is not generalized to the whole cardiovascular system.

Our study was not designed to investigate the mechanisms potentially responsible for the differential effects of RDN on MSNA/SSNA. However, we can hypothesize that baroreflex mechanisms are involved, given the evidence that (1) in physiological as well as in pathological conditions, reflexes stemming from arterial baroreceptors modulate MSNA but not SSNA [4, 16, 20, 21]; (2) an impairment in baroreflex modulation of MSNA has been documented in RHT [1]; and (3) RDN has been shown to improve baroreflex control of MSNA [23].

Other results of our study deserve to be briefly discussed. One, in our RHT patients, we did not find any quantitative or, in some instances, qualitative relationships between the BP reduction associated with RDN and the concomitant MSNA inhibition, which may suggest that the BP lowering effects of the intervention are not necessarily dependent on its sympathomodulatory effects. This finding, which has recently been confirmed by the results of a meta-analysis we performed examining the MSNA effects of RDNA in 11 published microneurographic studies for more than 400 patients [24], may suggest that mechanisms other than sympathetic ones participate in the BP lowering effects of RDN. A likely mechanism is the deactivation of the renin-angiotensin system, and thus the reduction of the circulating angiotensin II plasma levels exerting central and peripheral sympathoexcitation [25], which has been shown to take place during the first months following the procedure [26].

Second, in our study heart rate did not show any significant change in the 3-month period following RDN. Since heart rate represents a marker of the sympathetic drive to the heart [27], this finding may suggest that, similarly to the SSNA, cardiac sympathetic influences are not affected by the procedure. However, taking into account the data recently collected in the context of the SPIRAL-HT-OFF MED study [28], which showed a significant reduction in 24-h heart rate in a large group of RHT after RDN, it appears to be likely that the small sample size of our study, together with the short follow-up time, might have affected the results related to the heart rate responses to RDN. A further potential confounder was the presence of drugs such as beta-blockers, which may have affected resting heart rate values [29]. Finally, a previous study based on the norepinephrine spillover technique has shown that RDN may reduce the net release of the adrenergic neurotransmitter from renal sympathetic nerves. It may be thus be concluded that, as shown in other clinical conditions including RHT [4], there is a close parallelism between the behavior of MSNA and renal sympathetic drive, which does not appear to be shared by the SSNA.

### Limitations

Our study has some limitations. These include the small sample size and the presence of antihypertensive drugs, which may have affected sympathetic activity. It should be emphasized, however, that the same drug classes employed in the RDN group were used in the control group, with no evidence of any sympathetic change. In addition, SSNA is not affected by antihypertensive drugs [4, 16, 29], a finding which makes very unlikely that there was any interference of antihypertensive pharmacological interventions with the results obtained.

## Conclusion

This study has two major new findings: (1) there is evidence that in patients with RHT, the sympathetic overactivity does not uniformly affect the sympathetic cardiovascular drive, because although MSNA is markedly increased, SSNA is in the normal range, and (2) RDN triggers a sympathetic deactivation, which occurs in the muscle but not in the skin vascular district. These differential effects of RDN may be because, while MSNA is under baroreflex mechanisms (which are activated by the RDN), SSNA is modulated by thermoregulatory and emotional mechanisms usually unaffected by RDN [14, 20, 21].

## Data Availability

The data that support the findings of this study are available from the corresponding author upon reasonable request.
